# Solventless, selective and catalytic oxidation of primary, secondary and benzylic alcohols by a Merrifield resin supported molybdenum(vi) complex with H_2_O_2_ as an oxidant†

**DOI:** 10.1039/c8ra05969a

**Published:** 2018-10-08

**Authors:** Jeena Jyoti Boruah, Siva Prasad Das

**Affiliations:** Department of Chemistry, School of Science, RK University Bhavnagar Highway, Kasturbadham Rajkot-360020 Gujarat India siva.spd@gmail.com siva.das@rku.ac.in jeena.jyoti@gmail.com jeena.jyoti@rku.ac.in +91-9678084296; Department of Chemistry, Moridhal College Moridhal, Dhemaji-787057 Assam India

## Abstract

Here, we have described the synthesis, characterization and catalytic activity of a dioxo-molybdenum(vi) complex supported on functionalized Merrifield resin (MR-SB-Mo). The functionalization of Merrifield resin (MR) was achieved in two-steps *viz.* carbonylation (MR-C) and Schiff base formation (MR-SB). The compounds, MR-C, MR-SB and MR-SB-Mo, were characterized at each step of the synthesis by elemental, SEM, EDX, thermal, BET and different spectroscopic analysis. The catalyst, MR-SB-Mo, efficiently and selectively oxidized a wide variety of alcohols to aldehydes or ketones using 30% H_2_O_2_ as an oxidant with reasonably good TOF (660 h^−1^ in case of benzyl alcohol). The catalyst acted heterogeneously under solventless reaction conditions and did not lead to over oxidized products under optimized conditions. The catalyst afforded regeneration and can be reused for at least five reaction cycles without loss of efficiency and product selectivity. A reaction mechanism for the catalytic activity of MR-SB-Mo was proposed and a probable reactive intermediate species isolated.

## Abstract 2

Synthesis of a Merrifield resin supported dioxomolybdenum(vi) complex. The compound was well characterized and it catalysed the oxidation of alcohols to aldehydes or ketones with high TOF.

## Graphical abstract

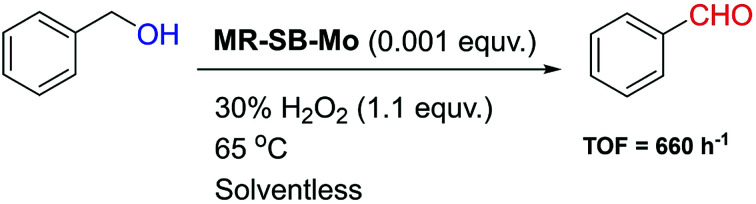

## 1. Introduction

The catalytic oxidation of alcohols is a basic transformation reaction, in synthetic organic chemistry in particular, used in the chemical and pharmaceutical industries, where aldehydes find wide application.^1^ Besides these, biologically the oxidation of alcohol to carbonyl offers a good contribution towards the degradation of fats during metabolism in humans (*e.g.*l-malate to oxaloacetate) which is part of the citric acid cycle.^2^ Due to the presence of a carbonyl group as an intermediate and final product in several natural products,^1^ fine chemicals^1^ and medicinally important components,^1^ there has been an upsurging interest in developing a variety of reagents and catalytic systems which has led to an improvement in the economic and environmentally benign synthetic methodology.

Traditional protocol for the oxidation of alcohol carried out by using stoichiometric inorganic oxidant such as permanganate, bromate, or Cr(vi) based reagents^3^ suffer from drawback such as large amount of heavy metal waste, generation of toxic by-products, difficulty in work up and requirement of larger amount of oxidizing agents.^3,4^ Moreover, in some cases, the oxidation reactions are performed under severe reaction conditions, such as high temperature and high oxygen pressure, in presence of environmentally undesired solvents, typically chlorinated hydrocarbons which are very toxic, corrosive and environmentally hazardous.^3,4*a*,5^ Therefore, search for alternative environmentally benign and safe protocols for the synthesis of different organic carbonyl compounds and their derivatives by alcohol oxidation continues unabated.

Although myriads of traditional user-friendly oxygen sources such as molecular oxygen, hydrogen peroxide, *tert*-butylhydroperoxide (TBHP) are used to eradicate such harmful waste for alcohol oxidation,^5^ but aqueous H_2_O_2_ constitutes a clean, waste avoiding, potentially green, and environmental-friendly ideal oxidant due to its cost effectiveness, easy availability, ecologically acceptable and it generates only water as the by-product.^6^ Also, the reaction parameters can be highly tuned by using H_2_O_2_ which play a vital role in oxidation reaction. We foresee that H_2_O_2_ and O_2_ (or air) will be complementary useful clean oxidants in practical chemical synthesis. Though molecular oxygen is an ideal oxidant, however, aerial oxidation has one or more of the major impacts such as difficult to control which result combustion and occasionally the reaction should have to be performed with a low conversion to circumvent from over oxidation limit their synthetic applications.^6*a*^ Besides these, although both the oxygen atom present in O_2_ may be employed for oxidation to meet the metrices for measuring the ‘greenness’ *i.e.* 100% atom efficiency, but in most of the reactions, only one oxygen atom is used and show only 50% atom efficiency, consequently to meet the requirement, the oxidation reactions often need certain reducing agents to arrest the extra oxygen atom during the reaction.^6*a*,7^ The rate of oxidation towards H_2_O_2_ induced oxidation reaction including sulfide, olefin and alcohol oxidation is relatively slow or negligible owing to its weak oxidative nature and as a consequence, it has to be activated by using a suitable catalysts.^1*b*,6*b*,8^ As a result, immense number of transition metal based catalytic systems such as vanadium,^9^ osmium,^10^ palladium,^11^ ruthenium,^12^ manganese,^13^ tungsten,^9*f*,*g*,14^ molybdenum,^9*f*,14*a*,*e*,*f*,15^ rhenium,^16^ cobalt,^11*c*,17^ copper,^11*c*,18^ and iron^19^ have been developed towards oxidation of alcohols. Though the transition metal ion complexes are reported to have their catalytic activity with high selectivity, good efficiency and reproducibility, however several catalytic processes associate with one or more of the disadvantages such as requirement of additives, use of halogenated solvents, homogeneous in nature, production of huge waste materials, corrosion to the industrial materials and some of them are deposited on the reactor wall as well as disrupting the environmental and ecological stability. These shortcomings are fetching increasingly conspicuous in the light of growing ecological awareness in recent years.^20^ Interestingly, over the recent years, a new class of catalyst has been developed which utilizes light for alcohol oxidation.^21^ These photocatalysts absorbs light (visible or UV) and coming under “Greener” approaches. However, majority of these protocols also suffered from drawbacks of the requirement of organic solvents and higher reaction time.

Therefore, constructing a safer, efficient and highly selective heterogeneous catalyst towards alcohol oxidation with H_2_O_2_ as the oxidant is considered to be the better choice. Apart from these, heterogenization of homogeneous catalysts^22^ by immobilization of active soluble catalysts on insoluble polymeric matrix offers improved stability, increased product selectivity, easy separation and recycling to the precious metal complexes.^22*a*,23^ In this regard, the use of Merrifield resin, which is a chloromethylated polystyrene cross-linked with divinyl benzene,^24^ is quite reasonable as the resin is readily availability, easy to handle, cheap, mechanically and chemically robust and most importantly able to undergo facile functionalization as well as provide high-loading capacity.^23*a*,25^

Keeping these facts into our account, we presented here the synthesis and characterization of a Merrifield resin supported dioxomolybdenum(vi) compound which acts as a heterogenous catalyst for the efficient and selective oxidation of alcohols to aldehydes or ketones. Interestingly, the protocol worked under solventless conditions with aqueous H_2_O_2_ as an oxidant and achieved the product selectivity with a wide range of substrates *viz.* primary, secondary and benzylic alcohols.

## 2. Experimental

### 2.1 Materials

The source of chemicals are given below: Merrifield resin (2% crosslinked, 2–4 mmol Cl per g of polymeric support), tetraethylammoniumchloride, 2-(aminomethyl)pyridine, 4-fluorobenzyl alcohol, 4-chlorobenzyl alcohol, 4-bromobenzyl alcohol, 4-methoxybenzyl alcohol, 4-hydroxybenzyl alcohol, diphenylmethanol, 1-phenylethanol, borneol, 2-octanol, 2-ethyl-1-hexanol, 1-butanol (Alfa Aesar, Mumbai, India), hydrogen peroxide (30%, 50% and 6%), sodium bicarbonate, dimethyl sulfoxide (DMSO), tetrahydrofurane (THF), methanol (MeOH), benzyl alcohol, 4-nitrobenzyl alcohol, cyclohexanol, cyclopentanol, menthol, 2-butanol, 1-octanol, 1-pentanol, 1-decanol (S D Fine-Chem Limited, Mumbai, India), silica gel (60–120 mesh) (Molychem, Mumbai, India). TLC plates (TLC Silica gel 60 F_254_) were purchased from Merck Limited (India). [MoO_2_(acac)_2_] was prepared by following a reported procedure.^26^ The other reagents and solvents were of commercially available reagent quality, unless otherwise stated.

### 2.2 Physical measurements

Elemental analysis of the compounds for C, H and N were carried out with the help of Perkin-Elmer 2400 series II CHN analyzer. The molybdenum content was determined by using atomic absorption spectroscopy (Thermo iCE 3000 series Atomic absorption spectrophotometer model analyst 200) and EDX analysis. Chlorine content was determined by EDX analysis. C, H and N content were also determined with the help of EDX analysis. IR spectra of the compounds were recorded in KBr pellet (4000–400 cm^−1^) using a Nicolet model 410 FTIR spectrophotometer. The UV-visible diffuse reflectance analysis of the samples were carried out with the help of a Hitachi U-3400 spectrophotometer equipped with an integrating sphere of 60 mm inner diameter using spectroscopic grade BaSO_4_ as a reference in the range of 240–800 nm. The SEM images of the samples were recorded by using the JEOL JSM-6390LV Scanning Electron Micrograph with an attached energy-dispersive X-ray detector. The dinitrogen adsorption/desorption measurements were carried out on a Quantachrome model Nova 4200e porosimeter at 77.3 K. The thermogravimetric analysis of the samples was done by using a SHIMADZU TGA-50 system under an atmosphere of nitrogen using an aluminium pan at a heating rate of 10 °C min^−1^. The powder X-ray diffraction (XRD) patterns were carried out by a Rigaku X-ray diffractometer (Miniflax, UK) using Cu Kα (*λ* = 0.154 nm) radiation over the 2*θ* range of 10–70°. The XPS measurements were conducted in a multi-probe system (Omicron Nanotechnology, Germany) equipped with a dual Mg/Al X-ray source and a hemispherical analyzer operating in constant analyzer energy (CAE) mode. The X-ray source (Mg Kα) was operated at 15 kV and 300 W. Charging effects were corrected by setting the binding energies of the adventitious C ls line at 284.8 eV. GC analysis was carried out on a CIC, Gas Chromatograph model 2010 using a SE-52 packed column (length 2 m, 1/8′′ OD) with a Flame Ionization Detector (FID), and nitrogen as the carrier gas (30 mL min^−1^). The ^1^H NMR spectra of the substrates were recorded with the help of Bruker, AVANCE 400 MHz spectrophotometer in CDCl_3_. Tetramethylsilane (TMS) or residual solvent peak was used as an internal standard.

### 2.3 Functionalization of the Merrifield resin (MR)

#### 2.3.1 Carbonyl functionalization of the MR (MR-C)

The MR was carbonyl functionalized by following a reported procedure with some modification.^27^ In the process, the MR was initially pre-swell in DMSO-THF (5 : 1) for 24 h at room temperature. Subsequently, the carbonyl functionalized polystyrene, MR-C was synthesized by mixing 1.0 g of MR with 0.50 g, 5.95 mmol sodium bicarbonate (NaHCO_3_) in 50 mL DMSO under reflux for 24 h. After cooling, the resultant polymer was filtered off, washed with water (3 × 10 mL) and methanol (3 × 10 mL), then dried under high vacuum.

#### 2.3.2 Synthesis of the Merrifield resin Schiff base support (MR-SB)

The Merrifield resin supported Schiff base was prepared by mixing MR-C with 2-(aminomethyl)pyridine in methanol. In the typical procedure, the MR-C (1.0 g) was pre-swell in 25 mL MeOH for 24 h at room temperature. Subsequently, 0.36 g, 3.32 mmol of 2-(aminomethyl)pyridine (1.5 equiv. of loaded carbonyl group) was added and refluxed the mixture for 48 h. The polymeric beads were slightly darkened from off-white color. After cooling, the solid was filtered off, washed several times by minimum amount of methanol, and then dried under high vacuum.

### 2.4 Synthesis of the Merrifield resin supported molybdenum(vi) complex (MR-SB-Mo)

The supported molybdenum(vi) complex was synthesized by reacting the MR-SB, and MoO_2_(acac)_2_ in MeOH-THF (1 : 1) under reflux condition. In the typical procedure, 1.0 g of MR-SB was swallowed in 25 mL MeOH-THF (1 : 1) for 24 h at room temperature. Subsequently, 0.82 g, 2.51 mmol of MoO_2_(acac)_2_ (1.5 equiv. of Schiff base loading) and 1.66 g, 10.02 mmol of tetraethylammoniumchloride (Et_4_NCl, 6 equiv. of Schiff base loading) in 5 mL MeOH was added and stirred the reaction mixture under reflux for 36 h. The reaction mixture was filtered and the solid was washed with MeOH (5 × 10 mL). The slightly brownish solid products were dried under high vacuum for 6 h and stored in dry place.

### 2.5 Catalytic activity of MR-SB-Mo

#### 2.5.1 General procedure for oxidation of alcohols to aldehydes or ketones

In the typical procedure, 2.5 mmol of the substrate (alcohol) was added to a 25 mL round bottom flask containing 2.75 mmol of 30% aqueous H_2_O_2_ and 5.6 mg of MR-SB-Mo (contain 0.0025 mmol of Mo). This maintained molar ratio of substrate : H_2_O_2_ : Mo = 1000 : 1100 : 1. The reaction mixture was stirred at 65 °C and progress was monitored by TLC and GC. After completion of the reaction, the solid catalyst was separated by filtration and the crude filtrate was extracted with ethyl acetate (3 × 5 mL). The combined extract was dried over anhydrous magnesium sulfate, filtered and removed the excess ethyl acetate under reduced pressure. This followed by column chromatography of the crude product on silica gel. The pure products were subjected to ^1^H NMR analysis for identification.

#### 2.5.2 Recyclability of the catalyst

The recyclability of MR-SB-Mo was studied by using benzyl alcohol as substrate. In the typical procedure, after completion of the reaction (as mentioned above) the solid catalyst was separated from the spent reaction mixture by filtration, washed with acetonitrile (3 × 5 mL) and dried *in vacuo*. The dried catalyst was then added to a fresh lot of reaction mixture maintain molar ratio of substrate : H_2_O_2_ : Mo at 1000 : 1100 : 1. The progress of the reaction was monitored by TLC and GC. After completion, the process was repeated so on for upto fifth reaction cycle.

## 3. Results and discussion

### 3.1 Synthesis

**Scheme 1:**
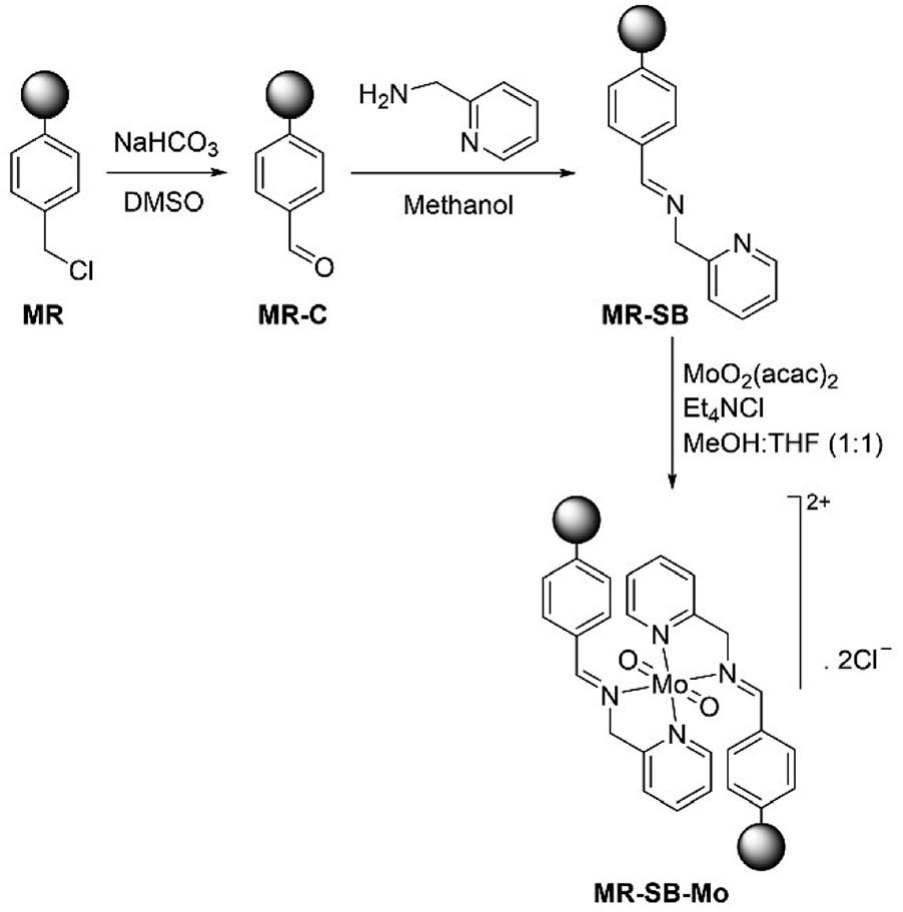
Synthesis of MR-SB-Mo. “
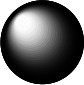
” represents polymeric support.

The synthetic pathway for the synthesis of the polymer-supported molybdenum(vi) compound, MR-SB-Mo is shown in Scheme 1. The carbonyl functionalization of MR to form MR-C was achieved by adopting a previously reported procedure.^27^ In the next step, the carbonyl group of MR-C was condensed with 2-aminomethylpyridine under reflux condition in methanol to form MR-SB. The molybdenum complex was formed by refluxing MR-SB, Et_4_NCl and MoO_2_(acac)_2_ in MeOH-THF (1 : 1) for 36 h. The Et_4_NCl provides the counter anion required for the formation of the molybdenum(vi) complex. In order to maximized the molybdenum loading, a series of reactions conducted under different reaction condition such as variation of solvent, reaction temperature and time, molar ratio of molybdenum : Schiff base, *etc.* The experiments revealed that 1.5 equivalent of MoO_2_(acac)_2_ with respect to Schiff base loading, MeOH/THF as solvent, under reflux condition and reaction time of 36 h were optimum for giving maximum molybdenum loading on MR-SB-Mo.

### 3.2 Characterization

The elemental analysis data for the compounds at each step of functionalization are shown below in Table 1. It is seen from the table that the compound MR-SB showed 4.68% of nitrogen which were absent in MR or MR-C. This indicates the formation of Schiff base by the reaction of carbonyl group of MR-C and 2-aminomethylpyridine. This corresponds to ligand (*i.e.*, 2-aminomethylpyridine) loading of 1.67 mmol g^−1^ of the polymer support. The value is consistent with the loss of the chlorine from the MR as evident from EDX analysis. From EDX analysis, it was also found that the amount of chlorine was decreased from 9.82% to 1.99% during carbonyl functionalization which indicates that nearly 80% of the chloromethylated groups were converted to carbonyl group in the process. The molybdenum loading in MR-SB-Mo was found to be 0.45 mmol g^−1^ of the polymeric support. This indicates that nearly half of the Schiff base ligands were coordinated with molybdenum (as two Schiff base molecules coordinated with one molybdenum center). Besides these, after metal incorporation, the chlorine content was increased from 1.69% (for MR-SB) to 4.60% (for MR-SB-Mo), which is due to the presence of the counter anion, Cl^−^ for charge neutralization as shown in the structure for MR-SB-Mo (Scheme 1). The magnetic susceptibility measurements of MR-SB-Mo showed the diamagnetic nature of the compound. This confirmed the +6 oxidation state of molybdenum centers.

**Table 1:** Chemical composition data for MR, MR-C, MR-SB and MR-SB-Mo

Compound	Data obtained from elemental analysis (%) (data obtained from EDX analysis (%))					Metal loading^a^ (mmol g^−1^ of polymer)
	C	H	N	Cl	Mo	
MR	83.20	6.98	—	—	—	—
	(83.13)	—		(9.82)		
MR-C	87.10	7.31	—	—	—	—
	(87.04)	—		(1.99)		
MR-SB	86.02	7.22	4.68	—	—	—
	(68.14)	—	(4.66)	(1.69)		
MR-SB-Mo	78.07	6.20	4.41	—	4.32^b^	0.45
	(78.21)	—	(4.36)	(4.60)	(4.28)	
					4.29^c^	

a Metal loading = (Observed molybdenum % × 10)/(atomic weight of molybdenum).

b Data obtained from AAS. “—” stands for not determined.

c Molybdenum content determined by AAS after 5^th^ reaction cycle.

**Fig. 1:**
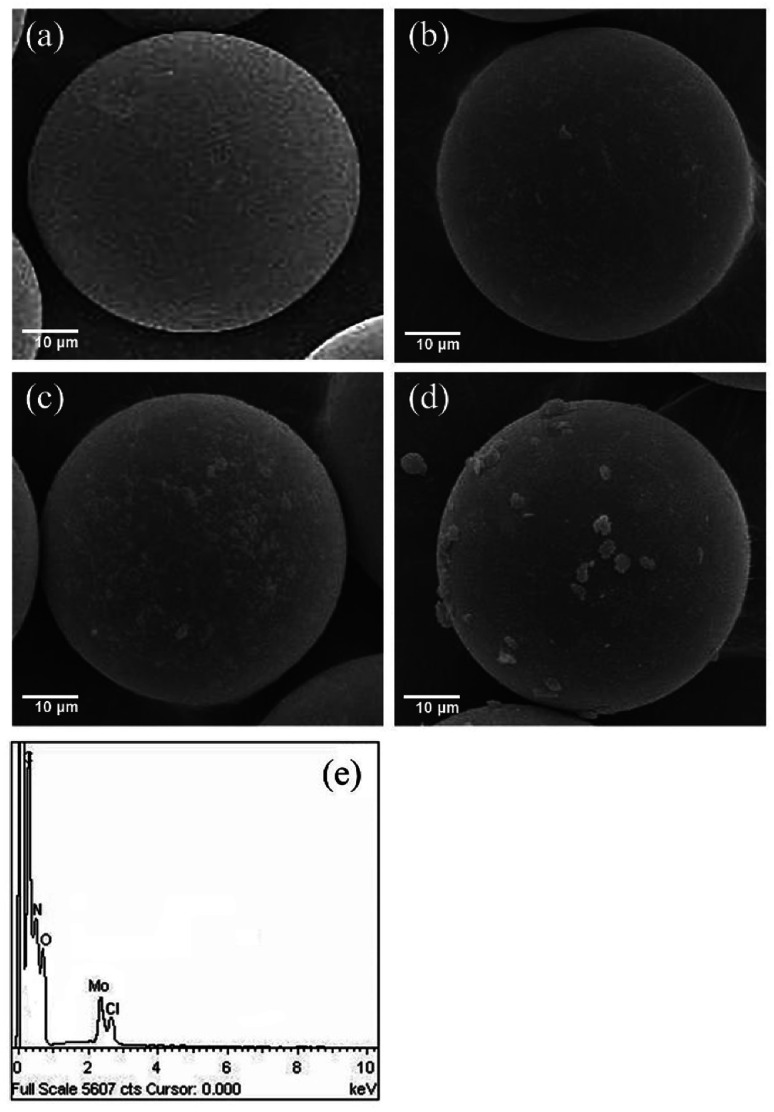
Scanning electron micrographs of (a) MR, (b) MR-C, (c) MR-SB, and (d) MR-SB-Mo. EDX spectra of (e) MR-SB-Mo.

The scanning electron micrographs (SEM) of the compounds obtained at different stages in the preparation of MR-SB-Mo are shown in Fig. 1. It is revealed from the micrograph that the virgin polymer beads, MR undergoes striking morphological changes to form MR-SB-Mo. The surface of the smooth and spherical beads of MR became lightly rough after carbonyl functionalization (MR-C, Fig. 1(b)) as well as Schiff base formation (MR-SB, Fig. 1(c)). The even roughening as seen in the images also suggest the uniform covalent functionalization on the surface of the MR beads. However, after molybdenum coordination, randomly oriented depositions were observed causing further roughening on the surface of the polymeric beads (Fig. 1(d)). The EDX spectra of MR-SB-Mo (Fig. 1(e)) showed the presence of carbon, nitrogen, oxygen, chlorine and molybdenum on the support.

**Fig. 2:**
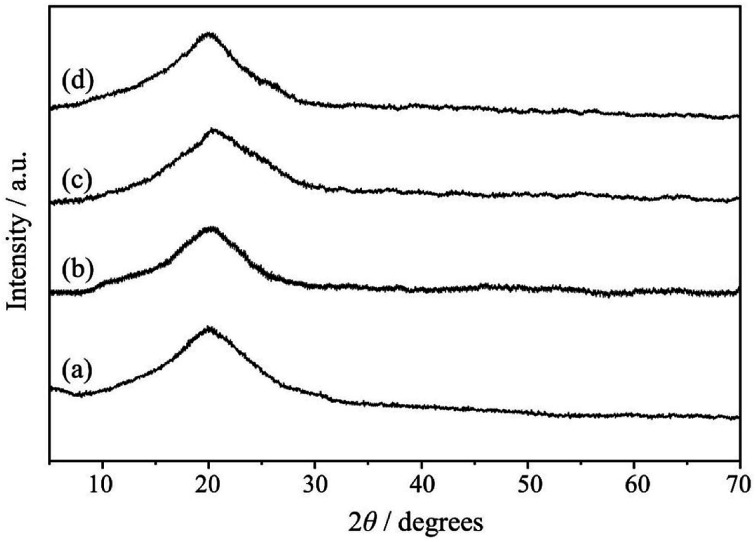
The XRD patterns of (a) MR, (b) MR-C, (c) MR-SB and (d) MR-SB-Mo.

The powder XRD patterns of MR, MR-C, MR-SB and MR-SB-Mo are shown in Fig. 2 and were recorded at 2*θ* values between 5 and 70°. A broad peak cantered at 2*θ* value of *ca.* 20° was observed in pristine MR. This type of diffraction pattern is characteristic for the PS-DVB resin.^28^ Similar to MR, almost identical diffraction patterns were observed in MR-C, MR-SB and MR-SB-Mo. This type of broad peak indicates that the developed catalyst and its precursors, *i.e.*, MR, MR-C and MR-SB are almost amorphous in nature. Moreover, the absence of sharp diffraction peaks in MR-SB-Mo indicated the amorphous nature of the attached MoO_2_^2−^ moiety. It is notable that during the synthesis of the MR-SB-Mo*via* different functionalization steps, there is no change in amorphousness of MR.

**Fig. 3:**
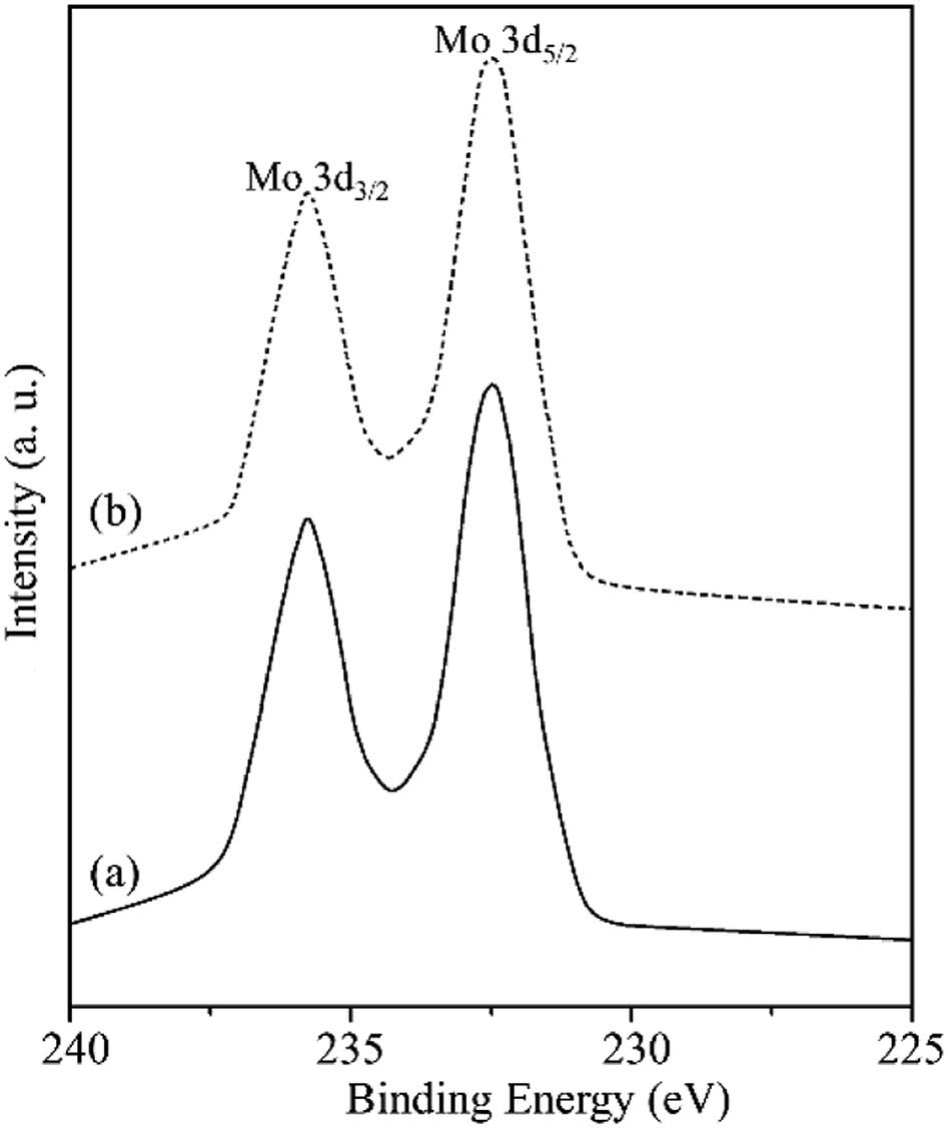
XPS Mo (3d_3/2_) and Mo (3d_5/2_) spectra of (a) MR-SB-Mo and (b) MR-SB-Mo after the 5^th^ reaction cycle.

In order to gain the electronic properties for the surface of MR-SB-Mo, XPS spectrum of the compound was recorded and presented in Fig. 3. From the figure it is seen that the spectrum displayed two well resolved peaks at 232.5 and 235.7 eV in the Mo (3d) region.^29^ On the basis of available literature reports,^29^ these peaks correspond to Mo (3d_3/2_) and Mo (3d_5/2_), respectively which is attributable to the molybdenum centers in +6 oxidation state, *i.e.*, diamagnetic. This diamagnetic behavior of MR-SB-Mo shown by XPS analysis is in agreement with the results obtained from magnetic susceptibility analysis.

The surface area, pore volume, and pore size of the synthesized compounds *viz.*MR, MR-C, MR-SB, and MR-SB-Mo, were investigated by N_2_ absorption and desorption measurements at liquid nitrogen temperature. The surface areas were measured by following the Brunauer–Emmett–Teller (BET) method^30^ and the pore volume was determined by following the Barrett–Joyner–Halenda (BJH) model in the nitrogen isotherms.^31^ The data are presented in Table 2. It is seen from the data that the surface areas, pore volumes and pore radius were decreased with increasing functionalization steps. This is because the functionalization may as well as metal loading blocked the pore of the polymeric beads. Similar types of observation were reported earlier with MR based catalysts.^32^ The nitrogen adsorption/desorption isotherms showed typical TYPE II adsorption (Fig. S1, ESI†) of an IUPAC standard^33^ which is the characteristics of macroporous or nonporous material.^34^

**Table 2:** The surface area, pore volume, and pore size of MR, MR-C, MR-SB, and MR-SB-Mo

Compound	*S*_BET_ a (m^2^ g^−1^)	*V*_tot_ b (cc g^−1^)	Pore radius (Å)
MR	12.3	0.14	51.5
MR-C	7.6	0.09	30.2
MR-SB	6.2	0.07	26.4
MR-SB-Mo	4.4	0.04	15.9

a BET surface area.

b Total pore volume.

**Fig. 4:**
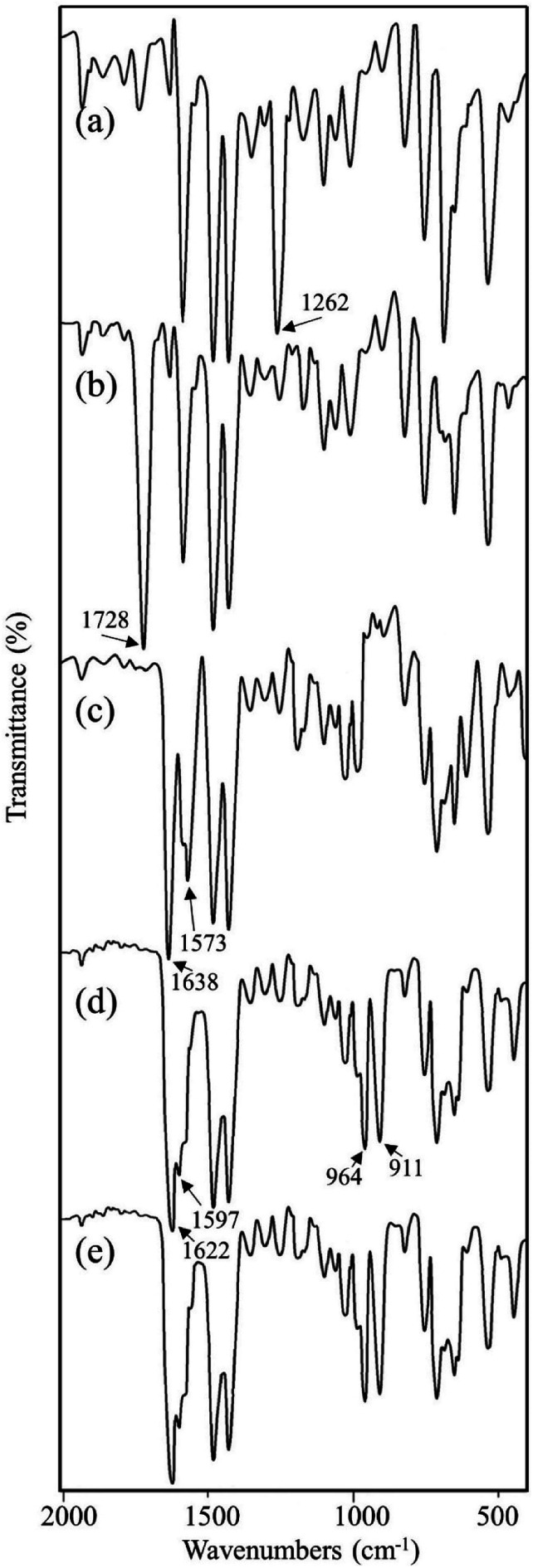
FTIR spectra for (a) MR, (b) MR-C, (c) MR-SB, (d) MR-SB-Mo, and (e) MR-SB-Mo after 5^th^ reaction cycle.

Vital information can be derived by comparing the infrared (IR) spectral data for the compounds at each synthetic step. After each synthetic step, characteristic new peaks may appear or shifted to a new position which confirmed the chemical transformations. The FT-IR spectra for the compounds are shown in Fig. 4 and important peaks are assigned in Table 3. On the basis of available reports, the change in intensity of the *ν*(C–Cl) peak is found to be the indicator of the functionalization of chloromethyl group.^32*a*,35^ It is evident by comparing the IR spectra of MR and MR-C that the strong peak appeared at 1262 cm^−1^ in MR which is attributed to *ν*(C–Cl)^32*a*,35*a*,*b*^ was disappeared (or significantly decreased the intensity) after carbonyl functionalization in MR-C. It is pertinent here to mention that after carbonyl functionalization the concentration of chloromethyl group in MR was dropped nearly 80%. Thus, this observation is in consistent with the data obtained from elemental analysis. Besides this, a strong peak appeared at 1728 cm^−1^ which is due to *ν*(C=O) of the aldehyde group.^36^ Additionally, two new weak intensity peaks appeared for *ν*(C–H)_aldehydic_ at 2826 and 2721 cm^−1^.^36*b*,*c*^ This further confirmed the conversion of chloromethyl group to aldehyde group.

**Table 3:** The IR spectral data for MR, MR-C, MR-SB, and MR-SB-Mo^a^

Compound	Peak position (cm^−1^)		Peak assignment
	IR	Raman	
MR	1262 (s)	1260 (w)	*ν*(C–Cl)
MR-C	1261 (vw)	—	*ν*(C–Cl)
	1728 (vs)	1730 (vw)	*ν*(C=O)
	2826 (m)	2822 (w)	*ν*(C–H)_aldehydic_
	2721 (m)	2726 (w)	
MR-SB	1261 (vw)	—	*ν*(C–Cl)
	1638 (vs)	1640 (m, sh)	*ν*(C=N)_imine_
	1573 (s)	1570 (m, sh)	*ν*(C=N)_py_
	609 (m)	610 (m)	Py(in-plane ring deformation)
	408 (m)	407 (m)	Py(out-of-plane ring deformation)
MR-SB-Mo	1263 (vw)	—	*ν*(C–Cl)
	1622 (vs)	1625 (m, sh)	*ν*(C=N)_imine_
	1597 (s)	1596 (m, sh)	*ν*(C=N)_py_
	964 (s)	962 (s)	*ν*(O=Mo=O)_asym_
	911 (s)	910 (s)	*ν*(O=Mo=O)_sym_
	640 (m)	645 (m)	Py(in-plane ring deformation)
	440 (m)	441 (m)	Py(out-of-plane ring deformation)

a Py, pyridine; vs, very strong; s, strong; m, medium; w, weak; vw, very weak; sh, shoulder; “—” stands for not seen.

In MR-SB, a new strong peak appeared at 1638 cm^−1^ which is attributable to *ν*(C=N)_imine_.^36*a*,37^ However, the peak at 1728 cm^−1^ due to *ν*(C=O) in MR-C was disappeared in MR-SB. This confirmed the condensation of aldehyde group with the amine group of 2-aminomethylpyridine to form an imine. Additionally, new medium to strong intensity peaks were appeared at 1573 (s), 609 (m), and 408 (m) cm^−1^ which can be assigned to *ν*(C=N)_pyridine_, Py(in-plane ring deformation), and Py(out-of-plane ring deformation), respectively of a pyridine moiety.^37,38^ Thus the IR spectral analysis clearly confirmed the formation of MR-SB as shown in Scheme 1. Interestingly, in MR-SB-Mo, new and strong peaks appeared at 964 and 911 cm^−1^ which were assigned for *ν*(O=Mo=O)_asym_ and *ν*(O=Mo=O)_sym_, respectively.^39^ Further the peak due to *ν*(C=N)_imine_ appeared at 1638 cm^−1^ of MR-SB was shift to 1622 cm^−1^ in MR-SB-Mo. In view of the existing literature, such lower shifting of *ν*(C=N)_imine_ peak confirmed the coordination of imine group with molybdenum(vi) center.^36*a*^ Apart from this, the peaks due to *ν*(C=N)_pyridine_, Py(in-plane ring deformation) and Py(out-of-plane ring deformation) were shifted to higher wave no after molybdenum(vi) coordination. This type of shifting suggest the coordination of pyridine with the metal center.^37,38*a*^ Thus the FTIR spectral analysis clearly showed the formation of MR-C, MR-SB and MR-SB-Mo.

**Fig. 5:**
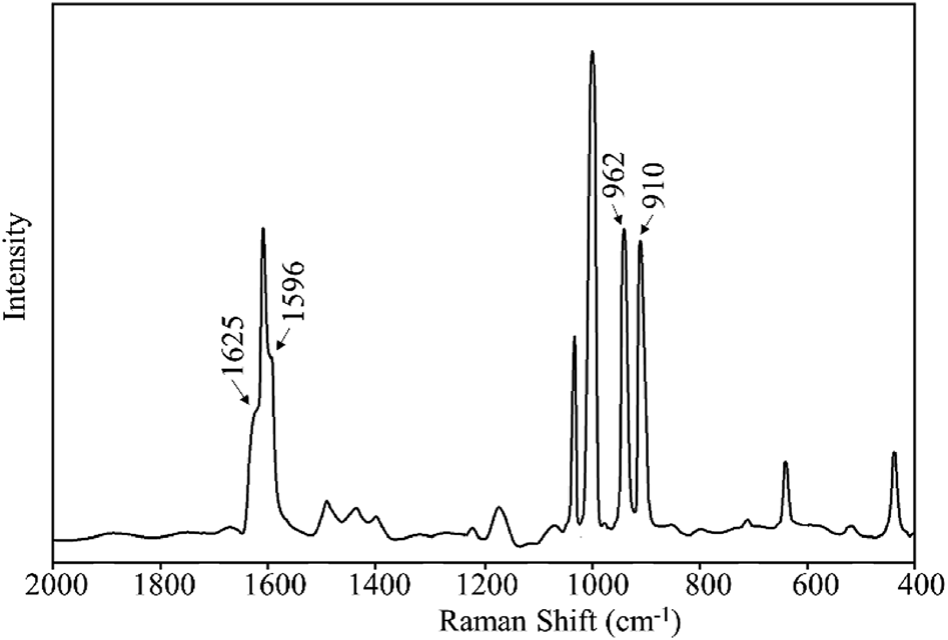
Raman spectrum for MR-SB-Mo.

The Raman spectral analysis further confirmed the formation of MR-C, MR-SB and MR-SB-Mo that shown by FTIR analysis. The spectral data of the samples are given in Table 3 and the spectrum for MR-SB-Mo is shown in Fig. 5. The strong peaks appeared commonly in each of the spectrum at *ca.* 1600 and 1000 cm^−1^ are attributed to the benzene skeletal vibrations arising from the polymeric backbone.^32*a*,40^ The weak peak appeared at 1260 cm^−1^ due to *ν*(C–Cl) mode of –CH_2_Cl group in MR was absent in MR-C.^40*a*^ This observation is in consistent with the results obtained from IR spectral analysis. The very weak peak appeared in the spectrum of MR-C at 1730 cm^−1^ is attributed to the *ν*(C=O) mode of aldehydic group. Similar to IR spectral analysis, this peak was vanished in the spectrum of MR-SB due to the Schiff base formation. The *ν*(C=N)_imine_ and *ν*(C=N)_pyridine_ peaks were appeared as medium intensity shoulder in the spectrum of MR-SB. However, after complex formation in MR-SB-Mo, the former peak was shifted to lower wave number and the latter was shifted to higher wave number that confirmed the coordination of the imine and pyridine moieties with the molybdenum(vi) center (through nitrogen atom). Further, the Raman spectrum of MR-SB-Mo displayed two strong peaks at 962 and 910 cm^−1^ for *ν*(O=Mo=O)_asym_ and *ν*(O=Mo=O)_sym_, respectively. These two peaks are characteristic of dioxomolybdenum(vi) moiety.^41^ Thus, the Raman spectral analysis confirmed the formation of MR-C, MR-SB and MR-SB-Mo.

The diffuse reflectance UV–visible spectra of MR-SB and MR-SB-Mo were recorded by taking BaSO_4_ as reference. The spectrum of MR-SB display two well resolved peaks at *ca.* 264 and 345 nm. The relatively higher energy peaks at 264 nm corresponds to the π → π* transition of the pyridyl ring, benzene ring of polymeric support and imine functional group.^37,42^ On the other hand, the lower energy peak at 345 nm may be assigned to n → π* transition of the pyridyl ring and imine functional group.^37,42^ Interestingly, in MR-SB-Mo, the peak for n → π* transition was shifted to *ca.* 331 nm. But the peaks for π → π* transition were remained unaffected after complex formation. This blue shift of n → π* transition indicate the coordination of molybdenum(vi) *via* the pyridyl and imine nitrogen.^37,42^ Beside these, there was no LMCT bands present in the UV-Vis spectrum of MR-SB-Mo. This is because those bands generally appear at higher concentration of metal complexes.

**Fig. 6:**
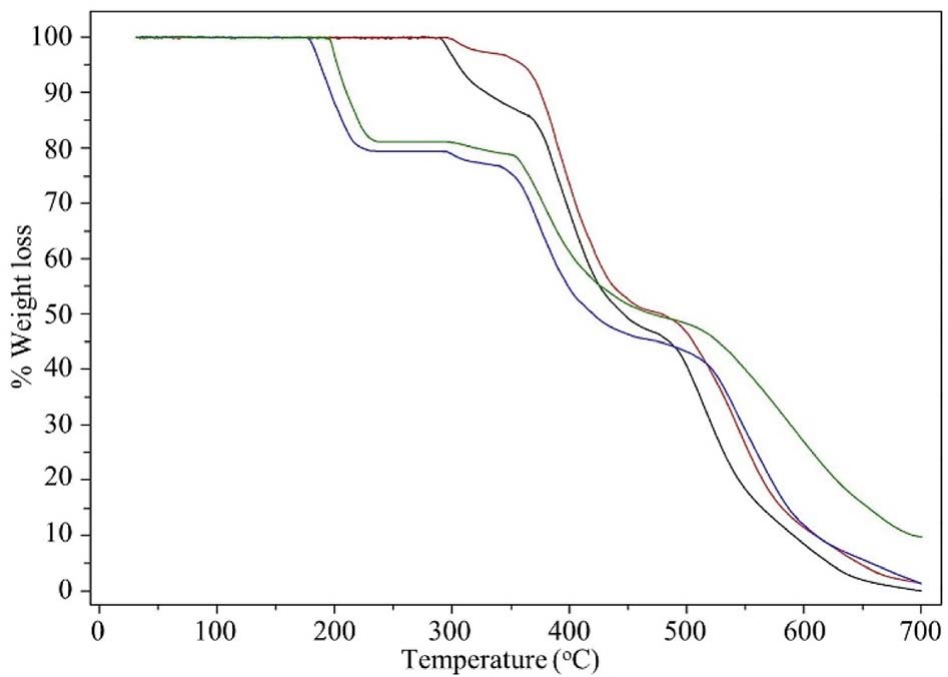
The TGA thermograms of MR (black), MR-C (red), MR-SB (blue), and MR-SB-Mo (green).

The thermal stability of the compounds was studied with the help of TGA-DTG analysis. The thermograms are shown in Fig. 6 and data are presented in Table 4. The thermograms showed multiple stage of thermal degradation for each of the compounds. The pristine polymer, MR showed three weight loss steps in the temperature range of 291–366, 366–477, and 477–700 °C with corresponding weight loss of 14.23, 39.53, and 46.24%, respectively. On the basis of available literatures data, the first step of decomposition is attributed to the loss of chloromethylated group and the subsequent steps are attributed to the decomposition of the cross-linked polystyrene backbone.^43^ The MR was completely decomposed at *ca.* 700 °C. Similar to MR, MR-C also showed three stage thermal decomposing. The thermogram of MR-SB provided vital information regarding the Schiff base loading on the support. The decomposition at *ca.* 177–230 °C with weight loss of 20.77% matched exactly with the data obtained from elemental analysis for Schiff base loading (20.53% Schiff base, based on N content). Further evidence about the loss of Schiff base ligand at this stage was confirmed by recording IR spectrum of the sample at this temperature which did not show the presence of characteristic peak for *ν*(C=N)_imine_. The subsequent decomposition steps are attributed to the decomposition of the polystyrene backbone. The thermogram of MR-SB-Mo showed four steps of thermal degradation at the temperature range of 195–234, 299–350, 350–486, and 486–700 °C with the corresponding weight loss of 18.86, 2.34, 29.90, and 39.27%, respectively. Interestingly, the compound never decomposed completely upto 700 °C which was due to the residual oxo-molybdenum species formed after complete decomposition of polymeric materials.^32*a*,44^ The thermal stability of upto 195 °C for MR-SB-Mo also provide additional evidence about the stability of the catalyst under different reaction temperatures.

**Table 4:** The TGA-DTG analysis data for MR, MR-C, MR-SB, and MR-SB-Mo

Compound	Temperature, ^o^C	Weight loss (%)
MR	291–366	14.23
	366–477	39.53
	477–700	46.24
MR-C	296–344	3.27
	344–480	46.74
	480–700	48.92
MR-SB	177–230	20.77
	294–340	2.46
	340–480	32.08
	480–700	43.47
MR-SB-Mo	195–234	18.86
	299–350	2.34
	350–486	29.90
	486–700	39.27

On the basis of the above analysis, a structure for MR-SB-Mo is proposed as shown in Scheme 1 where a dioxomolybdenum(vi) species is anchored to the support *via* coordination with the nitrogen atom of imine and pyridine group.

### 3.3 Catalytic activity

#### 3.3.1 Oxidation of alcohols to aldehydes or ketones catalyzed by MR-SB-Mo

The catalytic oxidation of alcohols to ketones (in case of secondary alcohols) or aldehydes (in case of benzylic and primary alcohols) by MR-SB-Mo using aqueous H_2_O_2_ as oxidant has been studied under solventless condition. The alcohols may be oxidized to aldehydes, ketones or carboxylic acids. The product selectivity depends upon the employed reaction conditions as discussed below.

In order to gain a standard reaction condition for product selectivity and better yield for oxidation of alcohols to ketones or aldehydes several reaction parameters such as solvents, amount of catalyst and oxidant, reaction temperature, *etc.* were screened using benzyl alcohol as model substrate.

In the initial investigation, the oxidation of benzyl alcohol was carried out with varying amount of 30% aqueous H_2_O_2_ as oxidant. We have conducted the reactions by keeping the molar ratios of benzyl alcohol : H_2_O_2_ at 1 : 0.5, 1 : 1.1, 1 : 1.5 and 1 : 2 and Mo: benzyl alcohol at 1 : 1000. The results are summarized in Table 5. The reaction at molar ratio of 1 : 0.5 was not completed even after 5 h [Table 5, entry 1]. So, we have increased the amount of H_2_O_2_ to 1 : 1.1, *i.e.*, slightly excess than the substrate. At this molar ratio, the reaction comfortably completed in 90 min with benzaldehyde as the sole product [Table 5, entry 2]. With a view to decrease the reaction time, we have further conducted the reactions at 1 : 1.5 and 1 : 2, but product selectivity was lost and formed 6% and 9% of benzoic acid, respectively [Table 5, entries 3 and 4]. Thus, the molar ratio of substrate : H_2_O_2_ at 1 : 1.1 is found to be the optimum condition for selectively oxidize benzyl alcohol to benzaldehyde.

**Table 5:** Optimization of reaction conditions catalyzed by MR-SB-Mo^a^

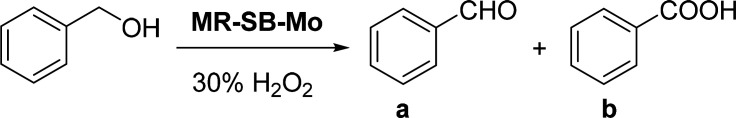								
Sl. no.	Molar ratio		Solvent	Temperature (°C)	Time (min)	Isolated yield (%)	Selectivity (%) *a* : *b*	TOF^b^ (h^−1^)
	Mo : S^c^	S : H_2_O_2_^c^						
1	1 : 1000	1 : 0.5	Solventless	65	300	47	100 : 0	94
**2**	**1** : **1000**	**1** **:** **1.1**	**Solventless**	**65**	**90**	**99**	**100** : **0**	**660**
3	1 : 1000	1 : 1.5	Solventless	65	70	97	91 : 6	831
4	1 : 1000	1 : 2.0	Solventless	65	60	98	89 : 9	980
5	1 : 1000	1 : 1.1	Water	65	90	87	100 : 0	580
6	1 : 1000	1 : 1.1	Acetonitrile	65	90	89	100 : 0	593
7	1 : 1000	1 : 1.1	Chloroform	65	90	24	100 : 0	160
8	1 : 1000	1 : 1.1	Dichloromethane	65	90	23	100 : 0	153
9	1 : 1000	1 : 1.1	Toluene	65	90	20	100 : 0	133
10	1 : 500	1 : 1.1	Solventless	65	80	98	100 : 0	368
11	1 : 100	1 : 1.1	Solventless	65	70	99	100 : 0	84
12	1 : 1000	1 : 1.1^d^	Solventless	65	150	96	100 : 0	384
13	1 : 1000	1 : 1.1^e^	Solventless	65	70	94	92 : 2	806
14	1 : 1000	1 : 1.1^f^	Solventless	65	300	97	100 : 0	194
15	1 : 1000	In air	Solventless	65	90	0	—	0
16	1 : 1000	In O_2_	Solventless	65	90	0	—	0
17	1 : 1000	1 : 1.1	Solventless	RT	300	98	100 : 0	196
18	1 : 1000	1 : 1.1	Solventless	50	120	99	100 : 0	495
19	1 : 1000	1 : 1.1	Solventless	90	90	91	100 : 0	607
20^g^	1 : 1000	—	Solventless	65	90	0	—	0
21^h^	—	1 : 1.1	Solventless	65	90	0	—	0
22^i^	1 : 1000	1 : 1.1	Solventless	65	90	53	91 : 9	353

a All reactions were carried out with benzyl alcohol as substrate (2.5 mmol), MR-SB-Mo (5.6 mg for 0.0025 mmol of Mo) and 5 mL solvent (unless otherwise indicated).

b TOF = (mmol of product)/[(mmol of catalyst) × (time)].

c ‘S’ stands for substrate.

d Reaction with 6% aqueous H_2_O_2_ as oxidant.

e Reaction with 50% aqueous H_2_O_2_ as oxidant.

f Reaction with 70% aqueous TBHP as oxidant.

g Reaction conducted with MR-SB-Mo but no added oxidant.

h Reaction conducted without MR-SB-Mo or blank reaction.

i Reaction conducted under optimum condition with SB-Mo (1.5 mg, 0.0025 mmol) as catalyst.

Keeping the molar ratio of substrate:H_2_O_2_ at 1 : 1.1, we have screened the oxidation of benzyl alcohol by using different solvents such as water, acetonitrile, chloroform, dichloromethane, toluene, *etc.* as well as under solventless condition. The highest activity in terms of TOF was found in solventless condition [Table 5, entry 2]. The reaction conducted in water decreased the TOF [Table 5, entry 5]. This may be due to dilution of the reaction mixture by the added water. In acetonitrile, the reactivity is not improved [Table 5, entry 6]. Further, reactions in chloroform and dichloromethane are very slow with poor TOF [Table 5, entries 7 and 8]. This may be due to the immiscibility of the oxidant with the solvents. Moreover, we do not intend to use halogenated solvents. Similar type of reactivity was also found while doing reaction in toluene [Table 5, entry 9]. The use of methanol and ethanol were avoided as these solvents may compete for oxidation. Indeed, the reactions were very slow and not completed (data not shown). Thus, solventless condition was found to be optimum for selectively oxidize benzyl alcohol to benzaldehyde.

The catalyst amount has a great impact on TOF. We have conducted reactions by keeping molar ratio of Mo : benzyl alcohol at 1 : 100, 1 : 500 and 1 : 1000. Each of the reactions were conducted by keeping benzyl alcohol: H_2_O_2_ at 1 : 1.1 and under solventless condition at 65 °C. There was decrease in reaction time with increased amount of catalyst but the TOF was significantly decreased [Table 5, entries 2, 10 and 11].

Reactions were conducted with 6%, 30% and 50% of aqueous H_2_O_2_ solutions [Table 5, entries 2, 12 and 13] at molar ratio of benzyl alcohol: H_2_O_2_ and Mo : benzyl alcohol at 1 : 1.1 and 1 : 1000, respectively under solventless condition at 65 °C. The slowest reaction and lowest TOF was found with 6% H_2_O_2_ whereas fastest was found with 50% H_2_O_2_ [Table 5, entry 13]. However, trace amount of carboxylic acid was formed with 50% H_2_O_2_, *i.e.*, loss of product selectivity. The slowest reaction with 6% H_2_O_2_ may be due to the dilution of the oxidant in the reaction mixture as was found in case of reactions in water as solvent [Table 5, entry 5]. Therefore, we have chosen 30% H_2_O_2_ as optimum for this reaction.

We have also screened the reaction with different types of oxidant such as 30% H_2_O_2_ (aqueous), *t*-butyl hydroperoxide (70% aqueous solution, TBHP), air and O_2_ balloon. The highest TOF was found with 30% H_2_O_2_ [Table 5, entry 2] and no reaction with air or O_2_ balloon under identical reaction conditions [Table 5, entries 15 and 16]. There were 3 to 4-fold increase in reaction time with TBHP [Table 5, entry 14] as an oxidant. Moreover, it was reported that TBHP generates butanol as byproduct.^45^ Interestingly, the use of H_2_O_2_ is advantageous as it is cheap, environmentally clean, easy to handle and water is the only byproduct.^6*a*–*c*,*e*,*g*^

The reaction temperature has a remarkable impact on rate of reaction. So, we have conducted reactions at different temperatures *viz.* room temperature, 50, 65, and 90 °C [Table 5, entries 2, 17–19]. The reactions were conducted under solventless condition by keeping molar ratio of benzyl alcohol : H_2_O_2_ (30%) : Mo at 1000 : 1100 : 1. It was found that with increasing reaction temperature, the TOF increases and reached a highest value at 65 °C. Thereafter, the TOF decreases which indicates that 65 °C is the optimum reaction temperature.

**Scheme 2:**
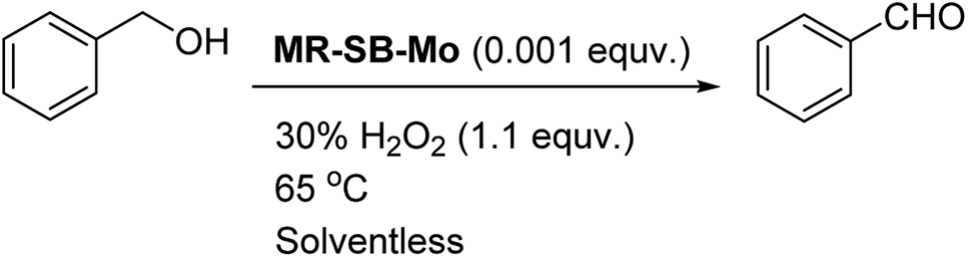
Optimum reaction condition for oxidation of alcohol catalyzed by MR-SB-Mo using 30% H_2_O_2_ as oxidant.

Thus the optimum reaction condition for the selective oxidation of benzyl alcohol to benzaldehyde was found to be substrate : H_2_O_2_ (30%) : Mo at 1000 : 1100 : 1 under solventless condition at 65 °C as shown in Scheme 2.

Having gained the optimal conditions, we explored the substrate scope of the newly developed catalyst, MR-SB-Mo under the optimum condition, for the selective oxidation of a wide range of alcohols such as primary, secondary and benzyl alcohols to their corresponding aldehydes or ketones. The results are summarized in Table 6. It is seen from the table that each of the substrate oxidized in high yields with reasonably good TOF. Besides this, under same reaction conditions, benzylic [Table 6, entries 1–9] and secondary alcohols [Table 6, entries 10–15] were found to be oxidize relatively at faster rate than the primary alcohols [Table 6, entries 16–20]. The beauty of the protocol is that no overoxidation to carboxylic acid took place with all the studied substrates. In case of substituted benzyl alcohols, different types of substituents such as –F, –Cl, –Br, –OMe, –OH and –NO_2_ well-tolerate during the oxidation process [Table 6, entries 2–7], some of which could be utilized for further derivation. One of the notable aspect of the developed catalytic system is its ability to oxidize benzyl alcohol to benzaldehyde at relatively higher scale (10 g scale) without losing the catalytic efficiency and product selectivity [Table 6, entry 1^*d*^] which provides its potential application towards commercial processes. It is pertinent here to mentioned that in a separate blank reaction using benzyl alcohol as substrate, *i.e.*, under identical optimum conditions without the added catalyst, the reaction did not progress within the stipulated time which indicate the active role of the catalyst in the oxidation processes (Table 5, entry 21). Similarly, the reaction was not successful without the added H_2_O_2_ (Table 5, entry 20).

**Table 6:** Oxidation of alcohols to aldehydes or ketones catalyzed by MR-SB-Mo using 30% aqueous H_2_O_2_ as oxidant^a^

Sl. no.	Substrate	Time (min)	Product	Isolated yield (%)	TOF^bb^ (h^−1^)
1	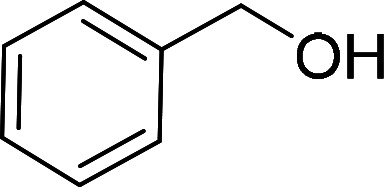	90	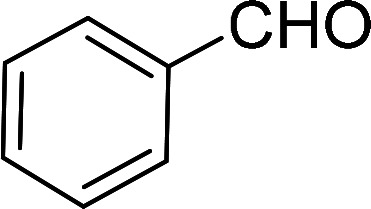	99	660
					653
					646
				98^c^	
				97^d^	
		90			
		90			
2^e^	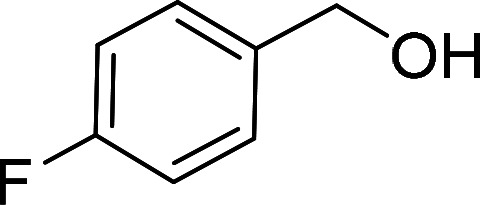	100	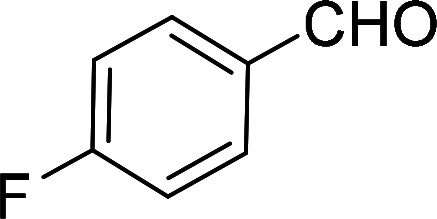	97	582
3^e^	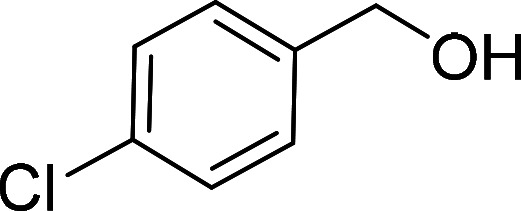	100	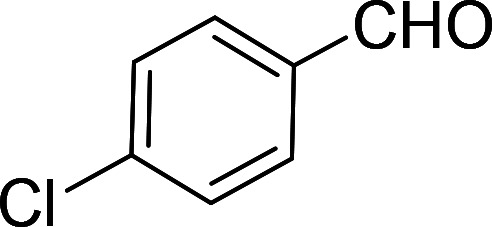	96	576
4^e^	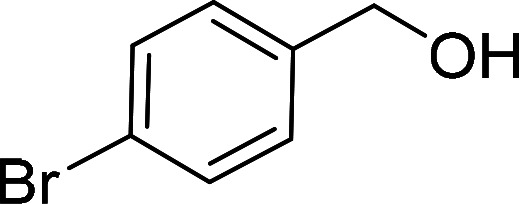	100	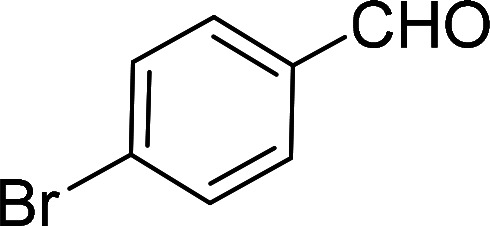	97	582
5^e^	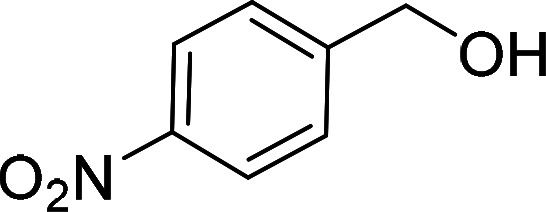	105	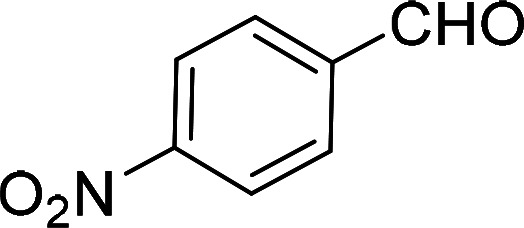	97	554
6^e^	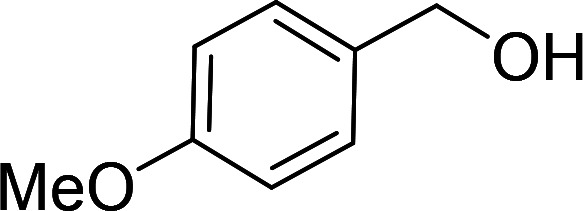	100	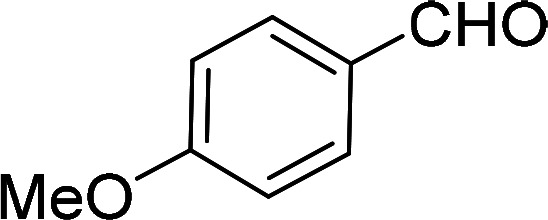	99	594
7	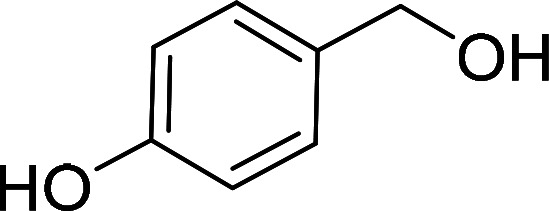	100	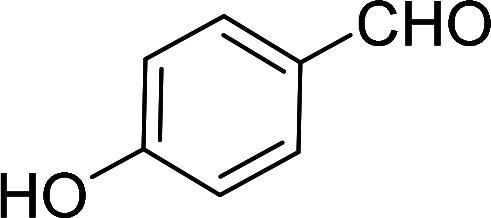	98	588
8	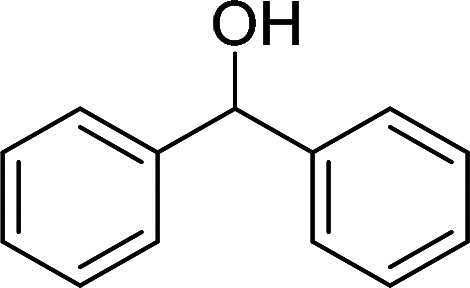	120	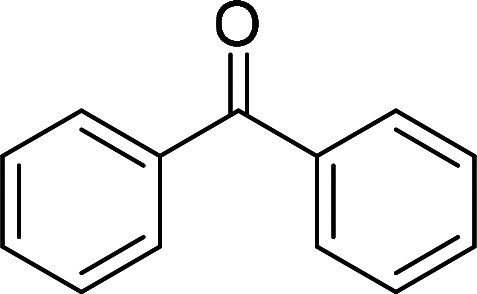	98	490
9	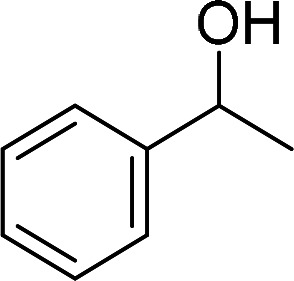	105	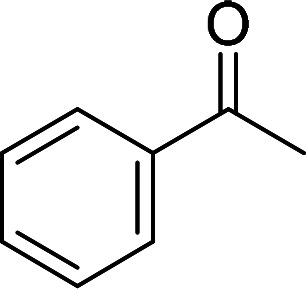	97	554
10	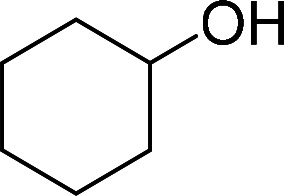	150	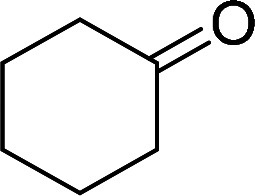	97	388
11	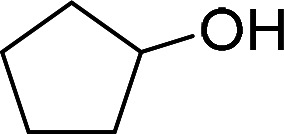	135	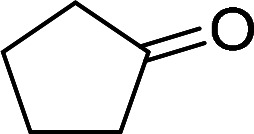	96	426
12	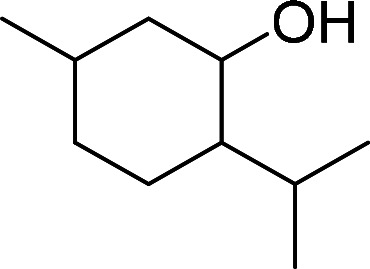	165	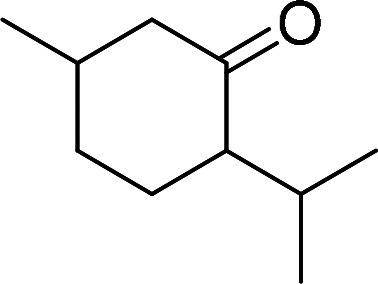	98	356
13^e^	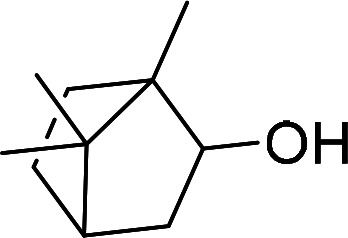	180	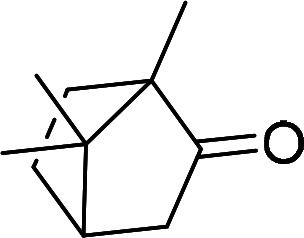	98	327
14	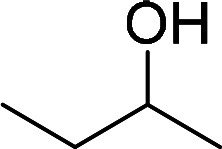	165	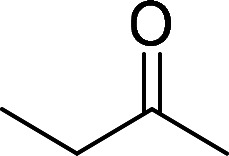	96	349
15	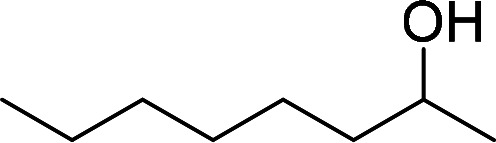	180	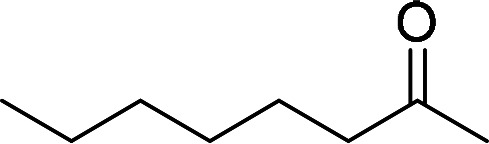	97	323
16	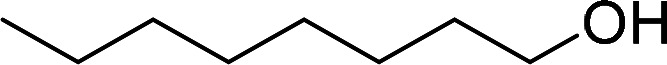	240	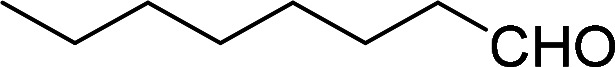	99	248
17	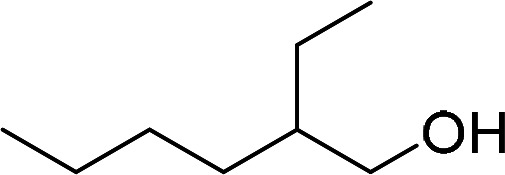	270	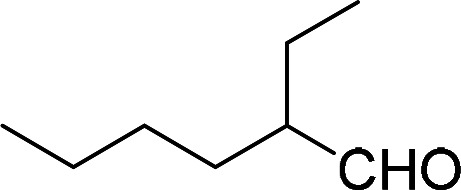	96	213
18	CH_3_(CH_2_)_3_CH_2_OH	240	CH_3_(CH_2_)_3_CHO	97	243
19	CH_3_(CH_2_)_8_CH_2_OH	270	CH_3_(CH_2_)_8_CHO	98	218
20	CH_3_(CH_2_)_2_CH_2_OH	240	CH_3_(CH_2_)_8_CHO	96	240

a Reaction conditions: unless otherwise stated, all reactions were performed solventless at 65 °C using 2.5 mmol of substrate, 5.6 mg of MR-SB-Mo (contain 0.0025 mmol of Mo) and 2.75 mmol of 30% aqueous H_2_O_2_.

b TOF=(mmol of product)/[(mmol of catalyst) × (time)].

c Yield at 5^th^ reaction cycle.

d Yield at 10 g scale reaction.

e Reaction conducted with 2 mL acetonitrile.

In order to check the advantage of synthesizing the heterogeneous catalyst, MR-SB-Mo, over homogeneous catalyst, a neat dioxomolybdenum complex, SB-Mo was synthesized (detailed synthetic procedure is in ESI†). The ligand for the neat complex was designed in such a way that it provides almost identical coordination environment that present in MR-SB-Mo. Moreover, the catalytic oxidation reaction was conducted under identical optimum condition using benzyl alcohol as substrate. From the reaction it was seen that the SB-Mo could reached upto TOF = 353 h^−1^ within the stipulated time period (Table 5, entry 22) and produced benzaldehyde along with benzoic acid (9%). Thus, under identical optimum condition the heterogeneous catalyst, MR-SB-Mo showed superior catalytic activity in terms of product yield as well as product selectivity over the homogeneous catalyst, SB-Mo.

In order to compare the catalytic activity of MR-SB-Mo over the reported catalyst, a separate comparison table containing catalytic activity of reported molybdenum-based catalyst towards oxidation of benzyl alcohol is given in ESI (Table S1†). From the table it is seen that MR-SB-Mo exhibit superior activity over the reported catalysts.

#### 3.3.2 Catalyst recycling

**Fig. 7:**
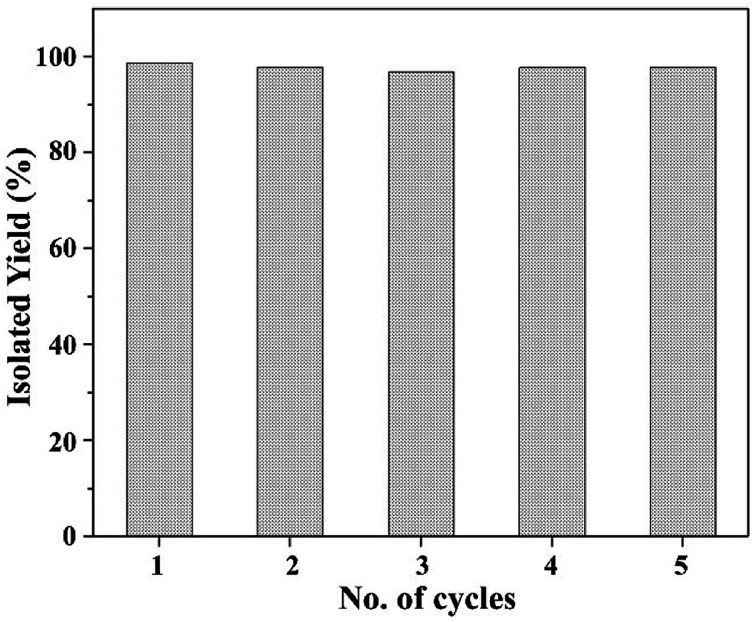
Catalyst recycling.

Recycling of the catalyst, MR-SB-Mo was examined under the optimum condition using benzyl alcohol as substrate. In the typical procedure, after completion of the reaction, the spent solid catalyst was separated from the reaction mixture by filtration, washed with acetonitrile, dried *in vacuo*, and used for the subsequent reaction cycles without any further treatment. Fig. 7 shows the result of the recycling experiments for five catalytic cycles. It was found that the catalyst efficiently and selectively oxidized benzyl alcohol to benzaldehyde without loss of its activity atleast upto fifth reaction cycles [Table 6, entry 1^*c*^]. No change in reaction yield and time were found even after the fifth reaction cycle. The FT-IR and XPS spectra of the spent solid catalyst recorded after fifth reaction cycle [Fig. 3(b) and 4(e)] were found to be identical with the fresh MR-SB-Mo. Moreover, the AAS analysis showed no significant loss of molybdenum content in the spent catalyst [Table 1]. These confirmed the stability and robustness of the catalyst in the oxidation process in presence of H_2_O_2_.

#### 3.3.3 Test for heterogeneity of the reaction

In order to test the leaching of the molybdenum complex from the polymer support, a separate experiment was carried out at optimum condition using benzyl alcohol as substrate. In the typical procedure, the reaction was run without the substrate, *i.e.*, benzyl alcohol for 90 min at 65 °C. Subsequently, the reaction mixture was filtered and separated the solid catalyst. To the filtrate, a fresh lot of benzyl alcohol and H_2_O_2_ were added and continued the reaction under identical condition for 90 min. No oxidation reaction was observed which is in agreement with the absence of metal leaching and pure heterogeneity of the catalytic process. Moreover, the AAS and ICP-OES elemental analysis showed no molybdenum in the filtrate.

#### 3.3.4 Mechanism of alcohol oxidation

**Scheme 3:**
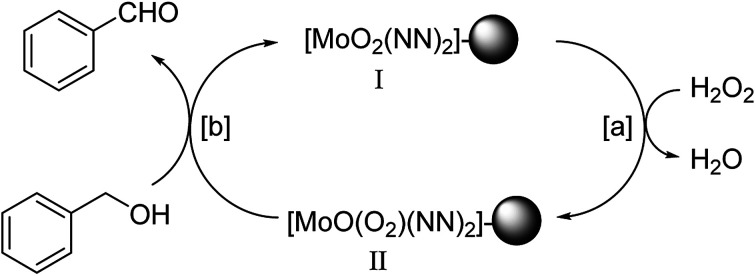
Proposed reaction mechanism for oxidation of alcohols using benzyl alcohol as representative. “NN” stands for the nitrogen coordination site for imine and pyridine group and “
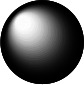
” represents polymeric support.

On the basis of our observations and the available literature data^14*f*,46^ for the oxidation reactions mediated by oxometal complexes, a mechanism is proposed in Scheme 3 for the catalytic activity of MR-SB-Mo. The catalyst, MR-SB-Mo which is a dioxomolybdenum(vi) complex, I is inactive as such in the oxidation process reacts with a hydrogen peroxide molecule *via* path [a] to form the peroxomolybdenum(vi) complex, II. The complex II is active in oxidation and oxidized the alcohol *via* path [b]. After oxidation, the active complex II became inactive and form complex I which is again reactivated by a new hydrogen peroxide molecule. This way the catalytic cycle is repeated. The formation of such active peroxomolybdenum(vi) complex, II from the inactive dioxomolybdenum(vi) complex, I by the reaction of hydrogen peroxide in the catalytic oxo-transfer process is well documented in literature.^14*f*,46^ Interestingly, in a separate experiment, we could isolate a peroxomolybdenum(vi) complex from MR-SB-Mo. In the typical experiment, MR-SB-Mo was treated with excess hydrogen peroxide in an ice-bath and the products were isolated by filtration, washed with acetonitrile and dried *in vacuo*. The original slightly brownish polymeric beads were changed into reddish color. The FTIR spectra for the reddish color beads is presented in Fig. S2(a) (ESI†) which shows the presence of characteristic peaks for *ν*(O–O), *ν*_sym_(Mo–O_2_) and *ν*_sym_(Mo–O_2_) in the vicinity of *ca.* 856 (s), 632 (m), and 514 (m) cm^−1^, respectively. These peaks are attributed to a peroxometal moiety.^32*a*,44^ Unfortunately, the isolated peroxo complex was not stable for prolonged period and gradually vanished the reddish color within two days. The FTIR spectra of the sample recorded at this stage showed the absence of peaks responsible for peroxometal moiety. But the IR spectrum was identical with the spectrum for MR-SB-Mo (Fig. S2(b), ESI†). This observation suggested that the MR-SB-Mo is converted into an active peroxometal moiety (may be II) by the reaction with the additional hydrogen peroxide which is revert back to its original form (MR-SB-Mo) after the oxidation reaction.

The change of substituents in the ligand environment of the metal complexes lead to a dramatic change in their catalytic activity. In this regard, the Hammett relation^47^ can be used to predict the catalytic activity of MR-SB-Mo. The substituent constant, *σ* of Hammett equation, for the substituents with (+)ve and (−)ve values indicate the electron withdrawing and electron releasing group, respectively. Thus, choosing substituents from the series could help to design a better catalyst for their activity. From the available literature report,^14*f*,47,48^ it is found that oxidation of alcohol by the peroxometal complexes take place by electrophilic attack of the peroxo moiety on the oxygen atom of the alcohol group (–OH). Thus, adding substituents with (+)ve *σ*-value in the ligand environment of MR-SB-Mo will increase the electrophilicity of the peroxo moiety which in return increase the catalytic activity of the catalyst. The catalyst, MR-SB-Mo can be substituted in its both the aromatic rings *viz.* phenyl or pyridyl ring. So, the synthesis of MR-SB-Mo with electron withdrawing substituents anticipated a higher reaction rate.

## 4. Conclusions

In summary, we have developed a heterogeneous molybdenum(vi) based catalyst using Schiff base functionalized Merrifield resin as support. The compound, MR-SB-Mo, served as an efficient catalyst for the selective oxidation of alcohols to aldehydes or ketones using 30% aqueous H_2_O_2_ as an oxidant, which is considered as a Green oxidant, under solvent-less condition. The catalyst showed recyclability atleast upto 5^th^ reaction cycles without loss of activity and product selectivity. The developed protocol for the oxidation of alcohols is simple and the products were isolated in highly pure form. On the basis of available literature reports (Table S1, ESI†), the catalyst is found to offer reasonably good catalytic activity and higher TOF. Thus, the protocol complies with the principles of “Green Chemistry” which is very important in concern with the current environmental prospects.

## Conflicts of interest

There are no conflicts of interest to declare.

## Supplementary Material

RA-008-C8RA05969A-s001
