# Supplementary material for: Solventless, selective and catalytic oxidation of primary, secondary and benzylic alcohols by a Merrifield resin supported molybdenum(vi) complex with H2O2 as an oxidant
Source: RSC Adv. 2018 Oct 8;8(60):34491–504. doi: 10.1039/c8ra05969a (PMC9086892; doi:10.1039/c8ra05969a)
Supplement: RA-008-C8RA05969A-s001 [file RA-008-C8RA05969A-s001.pdf]

Electronic Supplementary Material (ESI) for RSC Advances.
This journal is © The Royal Society of Chemistry 2018

Electronic Supplementary Information (ESI)

Solventless, Selective and Catalytic Oxidation of Primary, Secondary and Benzylic Alcohols by Merrifield Resin Supported Molybdenum(VI) Complex with H_2O_2 as an Oxidant

Jeena Jyoti Boruah*[a,b,c] and Siva Prasad Das*[a,c]

[a] *Department of Chemistry, School of Science, RK University, Bhavnagar Highway, Kasturbadham, Rajkot-360020, Gujarat, India.*
[b] *Department of Chemistry, Moridhal College, Moridhal, Dhemaji-787057, Assam, India.*
[c] *Both the authors contributed equally to this work.*

* Corresponding Authors:
E-mail: siva.spd@gmail.com, siva.das@rku.ac.in,
jeena.jyoti@gmail.com, jeena.jyoti@rku.ac.in
Phone: +91-9678084296

Synthesis of homogeneous dioxomolybdenum(VI) Schiff base complex (SB-Mo):

In order to compare the reactivity of the heterogeneous **MR-SB-Mo** with a homogeneous catalyst, a neat dioxomolybdenum(VI) Schiff base complex was synthesized. The ligand for the neat complex was designed in such as way that it provides almost identical coordination environment present in **MR-SB-Mo**.

The homogeneous neat complex was synthesized in two steps as follows.

(a) Synthesis of the ligand

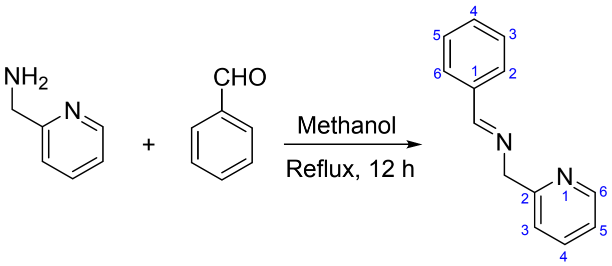

In the typical procedure, the Schiff base ligand was synthesized by refluxing 2.00 g, 18.5 mmol of 2-(aminomethyl)pyridine and 1.96 g, 18.5 mmol of benzaldehyde in 20 mL methanol for 12 h. 1-2 drops of glacial acetic acid was added as catalyst. After completion of the reaction, methanol was removed under reduced pressure and the solid obtained as such was washed thrice with hexane. Finally, the product was dried *in vacuo* (3.58 g, 18.2 mmol, 98.4%). ^{1}H NMR (300 MHz, $CDCl_3$): 5.05 (s, 1 H, -C*H_2*-), 7.10-7.21 (m, 2 H, Py-$H_{3,5}$), 7.52-7.61 (m, 3 H, Ph-$H_{3,4,5}$), 7.61-7.75 (m, 3 H, Py-H_4 and Ph-$H_{2,6}$), 8.61 (m, 1 H, Py-H_6), 8.76 (s, 1 H, -C*H*=N-) ppm. ^{13}C{^{1}H} NMR (75 MHz, $CDCl_3$): 65.9, 121.1, 121.9, 128.6, 129.7, 131.3, 135.8, 136.8, 148.7, 160.3, 162.4 ppm. Elemental analyses for $C_{13}H_{12}N_2$ calculated: C, 79.56; H, 6.16; N, 14.27%; found: C, 79.42; H, 6.28; N, 14.05%.

(b) Synthesis of the neat complex, SB-Mo

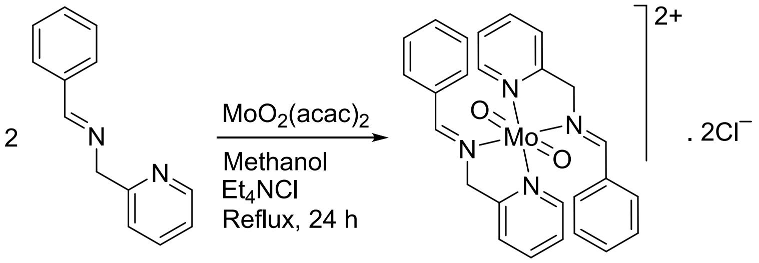

1.00 g, 5.1 mmol of the Schiff base ligand and 0.85 g, 2.6 mmol of $MoO_2(acac)_2$ were dissolved in 20 mL methanol. To this, 1.69 g, 10.2 mmol of Et_4NCl (2 equiv. of Schiff base) dissolved in 5 mL of methanol was added. The resulting solution was refluxed for 24 h. After completion of the reaction, the methanol was removed under reduced pressure and the solids were washed thrice with cold dichloromethane:acetonitrile (2:1). Finally, the product was washed thrice with hexane and dried *in vacuo* (1.12 g, 1.9 mmol, 73.1%). ^{1}H NMR (300 MHz, $CDCl_3$): 5.08 (s, 2 H, -C*H_2*-), 7.18-7.31 (m, 4 H, Py-$H_{3,5}$), 7.51-7.63 (m, 6 H, Ph-$H_{3,4,5}$), 7.63-7.76 (m, 6 H, Py-H_4 and Ph-$H_{2,6}$), 8.74 (m, 2 H, Py-H_6), 8.98 (s, 2 H, -C*H*=N-) ppm. ^{13}C{^{1}H} NMR (75 MHz, $CDCl_3$): 66.2, 122.3, 123.1, 128.8, 129.9, 131.1, 136.5, 137.2, 150.3, 161.8,

165.7 ppm. MS (ESI+, m/z) calculated for $C_{26}H_{23}MoN_4O_2$ $[M - H]^+$ m/z = 521.1, found 521.1. Elemental analyses for $C_{26}H_{24}Cl_2MoN_4O_2$ calculated: C, 52.81; H, 4.09; N, 9.47; Mo, 16.23%; found: C, 52.58; H, 3.89; N, 9.34; Mo, 16.08%.

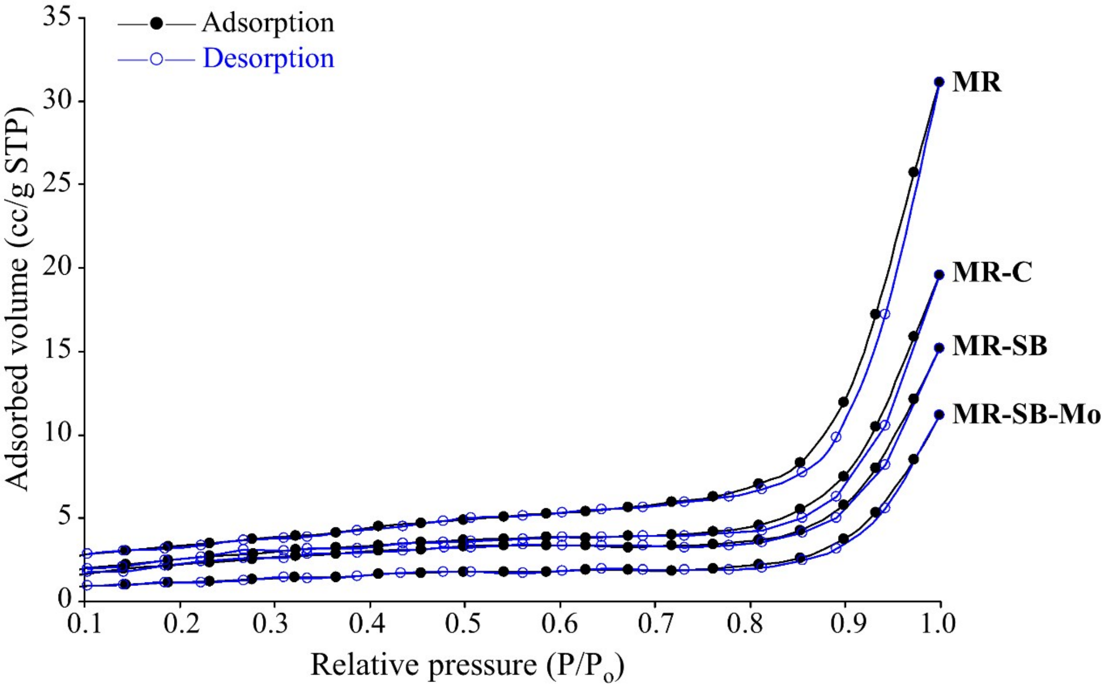

Fig. S1 The N_2 adsorption–desorption isotherms of **MR**, **MR-C**, **MR-SB** and **MR-SB-Mo**.

Isolation of peroxomolybdenum(VI) complex from MR-SB-Mo:

The peroxomolybdenum(VI) complex was formed by treating the **MR-SB-Mo** with excess 30% H_2O_2. In the typical procedure, 0.20 g of solid **MR-SB-Mo** was kept in an ice-bath at temperature below 4 °C. Subsequently, 0.50 ml of 30% H_2O_2 was added to it and allowed the reaction mixture to stand for 4 h (below 4 °C) with occasional stirring. During this time, the polymeric beads became reddish in color. The solid compound was separated by filtration, washed repeatedly with acetonitrile (3 × 3 ml), dried in *vacuo* for 8 h and stored in cool and dry place. The IR spectra shown below were recorded after drying (a) and two days later (b).

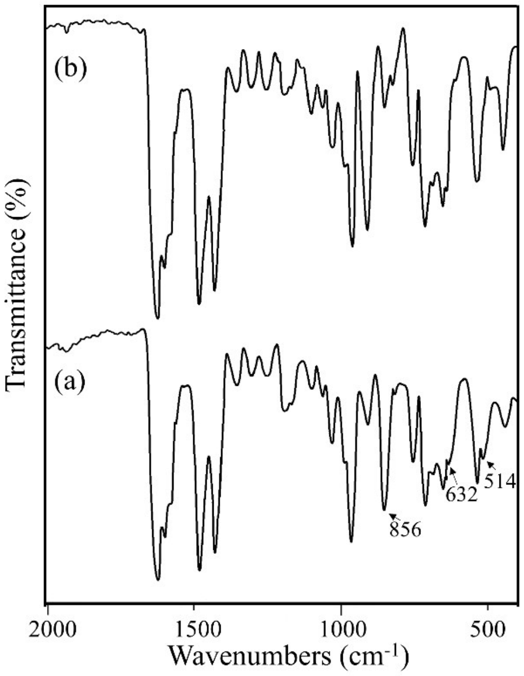

Fig. S2 FT-IR spectra of isolated peroxomolybdenum(VI) complex after (a) drying and (b) two days.

Table S1 Comparison of catalytic activity for the reported molybdenum-based catalysts towards oxidation of benzyl alcohol.

Sl. No.	Catalyst	Oxidant	TON	TOF (h^{-1})	Ref.
1	$CpMo(CO)_3(C{\equiv}CPh)$	H_2O_2	396	49.5	[1]
2	$H_5PV_2Mo_{10}O_{40}$	O_2	405	25.3	[2]
3	PMo_{11}/ Al_2O_3	H_2O_2	1125	47	[3]
4	PMA/VAMO	H_2O_2	24		[4]
5	PyC_4	H_2O_2	323	190	[5]
6	Polyaniline-$MoO_2(acac)_2$	O_2	29	2.4	[6]
7	$PPh_4[MoO(O_2)_2(HPEOH)]$	H_2O_2	630	26.25	[7]
8	$[MoO(O_2)_2QO][PPh_4]$	H_2O_2	840	56	[8]
9	$[(MoO_2)_2(slsch)(H_2O)_2]$	H_2O_2	431	144	[9]
10	$MoO_2(acac)_n$-NAP-MgO	O_2	16.2	1.35	[10]
11	$[MoO(O_2)_2(TEDA)_2]$	H_2O_2	96	16	[11]
12	CPS-$[MoO_2(ALGL)_2]$	O_2	100		[12]
13	$Ph_3P(CH_2)_2PPh_3[MoO(O_2)_2(C_2O_4)].2H_2O$	H_2O_2	49	8	[13]
**14**	**MR-SB-Mo**	$\mathbf{H_2O_2}$	**990**	**660**	**This work**
**15**	**SB-Mo**	$\mathbf{H_2O_2}$	**530**	**353**	**This work**

References

1. A. V. Biradar, M. K. Dongare and S. B. Umbarkar, *Tetrahedron Lett.*, 2009, **50**, 2885–288.
2. G. Maayan, B. Ganchegui, W. Leitner and R. Neumann, *Chem. Commun.*, 2006, 2230–2232.
3. A. Patel and S. Pathan, *Ind. Eng. Chem. Res.*, 2012, **51**, 732–740.
4. H. G. Manyar, G. S. Chaure and A. Kumar, *J. Mol. Catal. A-Chem.*, 2006, **243**, 244–252.
5. G. Ming-Lin and L. Hui-Zhen, *Green Chem.*, 2007, **9**, 421–423.
6. S. Velusamy, M. Ahamed and T. Punniyamurthy, *Org. Lett.*, 2004, **6**, 4821-4824.
7. N. Gharah, S. Chakraborty, A. K. Mukherjee and R. Bhattacharyya, *Inorg. Chim. Acta*, 2009, **362**, 1089–1100.
8. S. K. Maiti, Surajit Banerjee, Alok K. Mukherjee, K. M. Abdul Malikc and R. Bhattacharyya, *New J. Chem.*, 2005, **29**, 554–563.
9. S. D. Kurbah, A. Kumar, I. Syiemlieh, M. Asthana, R A. Lal, *Inorg. Chem. Commun.*, 2017, **86**, 39–43.

10. M. L. Kantam, J. Yadav, S. Laha, B. Sreedhar and S. Bhargava, *Adv. Synth. Catal.*, 2008, **350**, 2575–2582.

11. Y. Luan, G. Wang, R. L. Luck, M. Yang and X. Han, *Chem. Lett.*, 2007, **36**, 1236-1237.

12. B. Gao, M. Wan, J. Men and Y. Zhang, *Appl. Catal. A-Gen.*, 2012, **439–440**, 156–162.

13. X.-Y. Shi and J.-F. Wei, *J. Mol. Catal. A-Chem.*, 2005, **229**, 13–17.
